# Correlated expression of MYBL2 and CCL5 defines an immunosuppressive microenvironment and predicts poor prognosis in intrahepatic cholangiocarcinoma

**DOI:** 10.1186/s12885-026-16219-4

**Published:** 2026-06-17

**Authors:** Junkai Ren, Sicheng Xu, Xing He, Huilin Ye, Wenbin Li

**Affiliations:** 1 Department of Biliary and Pancreatic Surgery, Sun Yat-sen Memorial Hospital, Sun Yat-sen University, Guangzhou, Guangdong 510120 China; 2 Guangdong Provincial Key Laboratory of Malignant Tumor Epigenetics and Gene Regulation, Sun Yat-sen Memorial Hospital, Sun Yat-sen University, Guangzhou, Guangdong 510120 China

**Keywords:** Intrahepatic cholangiocarcinoma, MYBL2, CCL5, Tumor-associated macrophages, Immune microenvironment, PD-1 blockade, Prognostic biomarker

## Abstract

**Background:**

The tumor immune microenvironment critically influences progression and therapeutic response in intrahepatic cholangiocarcinoma (ICC). MYBL2 drives tumor aggressiveness, while CCL5 regulates immune-cell recruitment; however, their combined impact on immune composition and PD-1 therapy response remains undefined.

**Methods:**

Tumor specimens from 127 ICC patients were assessed by immunohistochemistry and whole-slide digital quantification. MYBL2 and CCL5 expression were classified into four combinatorial phenotypes. Associations with immune-cell infiltration, survival, and treatment response were evaluated using correlation analysis, Kaplan–Meier curves, Cox regression, and PD-1 interaction analyses. Seventy patients received adjuvant PD-1 therapy.

**Results:**

MYBL2 and CCL5 expression were positively correlated (*r* = 0.38, *p* < 0.001). Higher CCL5 levels were associated with increased CD163⁺ TAM density (*r* = 0.31, *p* < 0.001) and a reduced CD8/CD163 ratio, indicating an immunosuppressive microenvironment. The MYBL2–CCL5 classification provided clear prognostic discrimination, with the Double-High subgroup showing the shortest OS and PFS. Among PD-1–treated patients, treatment benefit varied markedly across phenotypes: the Double-Low group achieved the greatest survival advantage, whereas the Double-High group showed minimal response, reflecting primary resistance. In multivariable analysis, Double-Low remained strongly protective compared with Double-High.

**Conclusion:**

The MYBL2–CCL5 co-expression phenotype identifies an immunosuppressive, TAM-dominant microenvironment and serves as a dual biomarker for both prognosis and PD-1 treatment sensitivity in ICC. This immunologic classification offers a practical framework for postoperative risk stratification and personalized immunotherapy decision-making.

**Supplementary Information:**

The online version contains supplementary material available at https://doi.org/10.1186/s12885-026-16219-4.

## Introduction

Intrahepatic cholangiocarcinoma (ICC) is the second most common primary liver cancer and remains associated with poor survival despite advances in surgery and systemic therapy. Effective biomarkers for postoperative risk stratification and treatment selection are still lacking [1, 2]. The tumor immune microenvironment (TME) strongly shapes ICC progression and therapeutic response, particularly the balance between cytotoxic CD8⁺ T cells and immunosuppressive CD163⁺ tumor-associated macrophages (TAMs) [3–5].

MYBL2 (also known as B-Myb), a member of the MYB proto-oncogene transcription factor family, is a key regulator of cell-cycle progression, proliferation, and cell survival [6]. It drives the G1/S and G2/M transitions by forming the MMB (MYBL2-MuvB) complex, which cooperates with FOXM1 to activate mitotic gene transcription, while also participating in DNA replication and maintenance of genome stability [7]. In multiple human cancers, including hepatocellular carcinoma, MYBL2 overexpression has been associated with aggressive biological behavior, such as enhanced cell proliferation, resistance to apoptosis, epithelial-to-mesenchymal transition, metabolic reprogramming, chemotherapy resistance, and poor clinical outcome [8, 9]. Emerging evidence further indicates that, beyond its canonical cell-cycle functions, MYBL2 can modulate immune-related pathways, including chemokine-associated signaling [10–12].

The chemokine CCL5 (RANTES) regulates immune-cell recruitment and frequently promotes protumorigenic myeloid infiltration, including TAMs [13]. CCL5 has also been widely implicated in the establishment of an immunosuppressive tumor microenvironment through its role in recruiting and shaping protumorigenic myeloid populations [13, 14]. In parallel, previous studies in other cancer types have suggested that MYBL2 may participate in chemokine-mediated immune regulation beyond its canonical role in cell proliferation, such as through the MYBL2–CCL2 axis in ovarian cancer and MYBL2-associated CCL15 signaling in bladder cancer [15, 16]. Oncogene-driven transcriptional programs can modulate chemokine networks, but whether MYBL2 is associated with CCL5 expression and related immune remodeling in ICC is unknown. In particular, the impact of combined MYBL2–CCL5 expression on immune-cell composition, clinical outcome and response to PD-1–based therapy has not been defined.

We therefore investigated the clinicopathologic, immunologic and prognostic significance of MYBL2–CCL5 co-expression in resected ICC and evaluated whether this phenotype identifies an immunosuppressive TME and distinct patterns of benefit from adjuvant PD-1 blockade.

## Materials and methods

### Patient cohort and clinicopathologic data

A total of 127 consecutive patients who underwent curative-intent hepatectomy for pathologically confirmed ICC at Sun Yat-sen Memorial Hospital between February 2019 and May 2024 were retrospectively included. Eligible patients had available formalin-fixed paraffin-embedded (FFPE) tumor tissue and complete clinicopathologic and follow-up data; those with distant metastasis at diagnosis or who died within 30 days after surgery were excluded. The study protocol was approved by the Institutional Ethics Committee of Sun Yat-sen Memorial Hospital (Approval No. SYSKY-2025-513-01).

Baseline variables included demographic characteristics, routine liver function and coagulation tests, serum tumor markers (AFP, CEA, CA19-9, CA125 and CA72-4), and pathological features such as tumor size and number, TNM stage, lymph-node and vascular invasion, capsule and perineural invasion, Ki-67 index and resection margin status. Details are summarized in Table 1. Variables with < 5% missing data were analyzed using complete-case analysis.

**Table 1:** Clinicopathologic features of ICC patients according to MYBL2 expression level

	Low (*N* = 63)	High (*N* = 64)	Overall (*N* = 127)	*P* value
Sex				0.080
Female	19 (30.2%)	30 (46.9%)	49 (38.6%)	
Male	44 (69.8%)	34 (53.1%)	78 (61.4%)	
Age				1
≤ 65	44 (69.8%)	44 (68.8%)	88 (69.3%)	
> 65	19 (30.2%)	20 (31.3%)	39 (30.7%)	
ALB				0.953
≤ 35	43 (68.3%)	45 (70.3%)	88 (69.3%)	
> 35	20 (31.7%)	19 (29.7%)	39 (30.7%)	
ALT				0.178
≤ 40	46 (73.0%)	54 (84.4%)	100 (78.7%)	
> 40	17 (27.0%)	10 (15.6%)	27 (21.3%)	
AST				0.178
≤ 40	46 (73.0%)	54 (84.4%)	100 (78.7%)	
> 40	17 (27.0%)	10 (15.6%)	27 (21.3%)	
ALP				0.053
≤ 120	36 (57.1%)	48 (75.0%)	84 (66.1%)	
> 120	27 (42.9%)	16 (25.0%)	43 (33.9%)	
GGT				0.226
≤ 60	20 (31.7%)	28 (43.8%)	48 (37.8%)	
> 60	43 (68.3%)	36 (56.3%)	79 (62.2%)	
TBil				0.145
≤ 17.1	42 (66.7%)	51 (79.7%)	93 (73.2%)	
> 17.1	21 (33.3%)	13 (20.3%)	34 (26.8%)	
APTT				0.119
≤ 35	60 (95.2%)	64 (100%)	124 (97.6%)	
> 35	3 (4.8%)	0 (0%)	3 (2.4%)	
AFP				0.208
≤ 10	62 (98.4%)	59 (92.2%)	121 (95.3%)	
> 10	1 (1.6%)	5 (7.8%)	6 (4.7%)	
CA19-9				0.529
≤ 37	36 (57.1%)	32 (50.0%)	68 (53.5%)	
> 37	27 (42.9%)	32 (50.0%)	59 (46.5%)	
CA125				0.953
≤ 35	43 (68.3%)	45 (70.3%)	88 (69.3%)	
> 35	20 (31.7%)	19 (29.7%)	39 (30.7%)	
CA72-4				**0.048**
≤ 6.7	44 (69.8%)	55 (85.9%)	99 (78.0%)	
> 6.7	19 (30.2%)	9 (14.1%)	28 (22.0%)	
CEA				0.124
≤ 5	35 (55.6%)	45 (70.3%)	80 (63.0%)	
> 5	28 (44.4%)	19 (29.7%)	47 (37.0%)	
Tumor Size				0.386
≤ 5	19 (30.2%)	25 (39.1%)	44 (34.6%)	
> 5	44 (69.8%)	39 (60.9%)	83 (65.4%)	
Tumor Number				0.615
Single	50 (79.4%)	54 (84.4%)	104 (81.9%)	
Multiple	13 (20.6%)	10 (15.6%)	23 (18.1%)	
Ki67				0.156
≤ 30	18 (28.6%)	27 (42.2%)	45 (35.4%)	
> 30	45 (71.4%)	37 (57.8%)	82 (64.6%)	
LN Metastasis				0.425
No	34 (54.0%)	29 (45.3%)	63 (49.6%)	
Yes	29 (46.0%)	35 (54.7%)	64 (50.4%)	
Perineural Invasion				0.415
No	25 (39.7%)	31 (48.4%)	56 (44.1%)	
Yes	38 (60.3%)	33 (51.6%)	71 (55.9%)	
Capsule Invasion				1.000
No	14 (22.2%)	14 (21.9%)	28 (22.0%)	
Yes	49 (77.8%)	50 (78.1%)	99 (78.0%)	
R0 Resection				**0.036**
No	19 (30.2%)	32 (50.0%)	51 (40.2%)	
Yes	44 (69.8%)	32 (50.0%)	76 (59.8%)	
Portal Invasion				0.953
No	43 (68.3%)	45 (70.3%)	88 (69.3%)	
Yes	20 (31.7%)	19 (29.7%)	39 (30.7%)	
IVC Invasion				0.206
No	56 (88.9%)	61 (95.3%)	117 (92.1%)	
Yes	7 (11.1%)	3 (4.7%)	10 (7.9%)	
Hepatic vein Invasion				0.868
No	50 (79.4%)	49 (76.6%)	99 (78.0%)	
Yes	13 (20.6%)	15 (23.4%)	28 (22.0%)	
Distant Metastasis				0.241
No	55 (87.3%)	60 (93.8%)	115 (90.6%)	
Yes	8 (12.7%)	4 (6.3%)	12 (9.4%)	
TNM Stage				0.934
Stage I–II	30 (47.6%)	29 (45.3%)	59 (46.5%)	
Stage III–IV	33 (52.4%)	35 (54.7%)	68 (53.5%)	

Of the 127 patients, 70 (55.1%) received postoperative adjuvant PD-1–based therapy after multidisciplinary discussion, mainly for high-risk pathological features including lymph-node metastasis, vascular invasion, multiple lesions, or narrow surgical margins. All PD-1–based treatments were administered in the postoperative adjuvant setting after curative-intent resection. The PD-1 inhibitors used included pembrolizumab, toripalimab, camrelizumab, sintilimab, and tislelizumab. Among these 70 patients, 30 received PD-1 inhibitor monotherapy, 22 received PD-1 inhibitor combined with chemotherapy, and 18 received PD-1 inhibitor combined with both chemotherapy and targeted therapy. The median number of PD-1 treatment cycles was 9.5 (range, 2–20). Patients were followed every 2–3 months during the first 2 years and every 3–6 months thereafter with contrast-enhanced CT or MRI and clinical assessment. Vital status and dates of death were obtained from hospital records and telephone follow-up; November 9, 2025 was the last follow-up date.

### Immunohistochemistry (IHC) staining

Formalin-fixed, paraffin-embedded ICC Sect.  (4 μm) were deparaffinized, rehydrated, and rinsed in PBST. Antigen retrieval was performed in EDTA buffer (pH 9.0) using heat-induced epitope retrieval. Endogenous peroxidase was quenched with 3% hydrogen peroxide for 15 min, and non-specific binding was blocked with 10% goat serum for 30 min.

Slides were incubated overnight at 4 °C with primary antibodies against MYBL2 (Proteintech, 8896-1-AP, 1:300), CCL5 (CST, 36467 S, 1:400), CD8 (Proteintech, 66868-1-Ig, 1:1000), and CD163 (CST, 93498 S, 1:1000). After washing, HRP-conjugated secondary antibodies were applied for 30 min. Staining was visualized with DAB and counterstained with hematoxylin, followed by dehydration, clearing, and mounting.

Whole-slide images (WSIs) were scanned and analyzed in QuPath (v0.5.1). Automated tissue segmentation and cell detection were applied across the entire tumor area without manual ROI selection, with necrosis, hemorrhage, stromal-free, and blank regions excluded by preset thresholds. Two pathologists, blinded to outcomes, reviewed and confirmed tumor regions; discrepancies were resolved by consensus.

Staining intensity was scored from 0 to 3, and the percentage of positive tumor cells was recorded. MYBL2 and CCL5 expression was quantified using the H-score method (0–300). For CD8⁺ and CD163⁺ immune cells, positive-cell density was calculated from whole-slide cell counts and tissue area extracted from WSIs. Immune-cell density (cells/mm²) was computed as:

$$Density=\frac{\mathrm{N}\mathrm{u}\mathrm{m}\:\mathrm{D}\mathrm{e}\mathrm{t}\mathrm{e}\mathrm{c}\mathrm{t}\mathrm{i}\mathrm{o}\mathrm{n}\mathrm{s}}{\mathrm{A}\mathrm{r}\mathrm{e}\mathrm{a}\:\left({{\upmu\:}\mathrm{m}}^{\mathrm{2}}\right)}\times 10^6$$

and reported as the mean density per case. To reflect the immune balance within the tumor microenvironment, the CD8⁺/CD163⁺ ratio was calculated as:

$$\frac{CD8^+}{CD163^+}ratio=\frac{CD8\;density(\frac{cells}{mm^2})}{CD163\;density\;(\frac{cells}{mm^2})+1\times10^{-6}}$$

with a small constant added to avoid division by zero in cases with absent CD163⁺ infiltration.

### Definition of expression groups

The H-scores of MYBL2 and CCL5 were dichotomized at the cohort median to define “High” and “Low” expression categories. Based on these cut-offs, patients were further stratified into four combined expression groups: Double-High: MYBL2-High / CCL5-High; MYBL2-High/CCL5-Low; MYBL2-Low/CCL5-High; Double-Low: MYBL2-Low/CCL5-Low. The Double-Low group served as the reference category in subsequent survival analyses.

### Correlation and immune cell analysis

Correlation coefficients were calculated to examine the relationships between MYBL2 and CCL5 H-scores and the densities of CD8⁺ and CD163⁺ immune cells. Positive correlations were interpreted as parallel increases in expression or infiltration. The correlations between CCL5 and immune-cell markers were specifically evaluated to explore potential patterns of immune-cell recruitment and macrophage polarization within the tumor microenvironment.

### Statistical analysis

Overall survival (OS) was defined as the interval from surgery to death or last follow-up, and progression-free survival (PFS) as the time from surgery to the first documented recurrence, metastasis, or death. Survival curves were generated using the Kaplan–Meier method and compared with the log-rank test. Prognostic factors for OS and PFS were evaluated with Cox proportional hazards models; variables with *p* < 0.10 in univariable analysis were entered into the multivariable model, and proportional hazards assumptions were checked using Schoenfeld residuals.

A prognostic nomogram for 1-, 2-, and 3-year OS was constructed from the final multivariable model. Model performance was assessed by the concordance index (C-index), 1,000-bootstrap calibration curves, and time-dependent ROC curves (timeROC package), and was compared with the AJCC 8th TNM staging system. Clinical utility was examined using decision-curve analysis (rmda package).

All analyses were performed in R (version 4.3.2) with the survival, rms, timeROC, rmda, and ggplot2 packages. To test whether the MYBL2–CCL5 phenotype modified the effect of PD-1 therapy, Cox models including PD-1 treatment status, the four-group classifier, and their interaction term were fitted and evaluated by likelihood-ratio test. Continuous variables were compared using the Student’s t test or Wilcoxon rank-sum test as appropriate, and categorical variables with the chi-square or Fisher’s exact test. Two-sided *p* values < 0.05 were considered statistically significant.

## Results

### Expression patterns of MYBL2, CCL5, CD8, and CD163 in ICC tissues

**Fig. 1:**
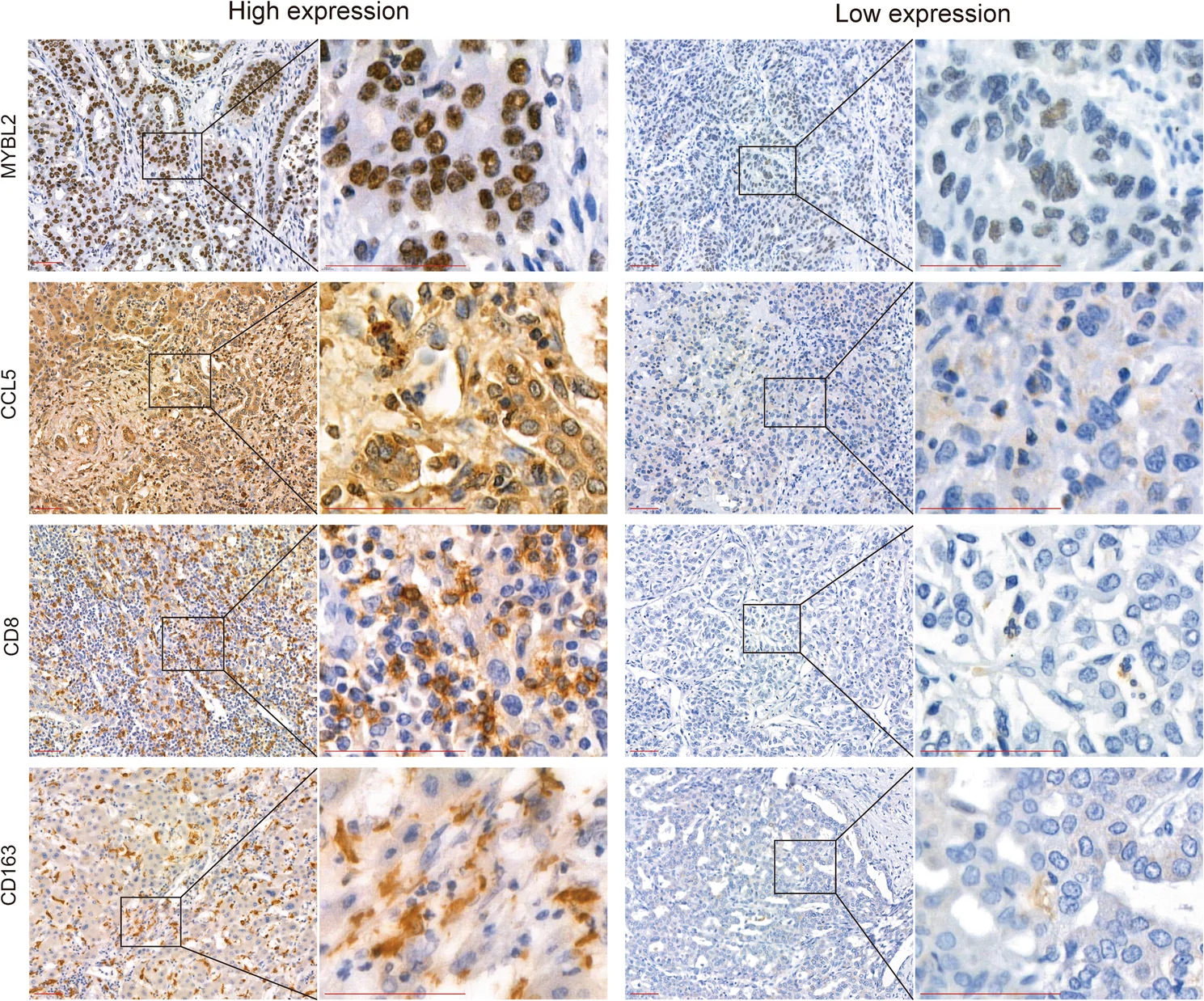
Representative immunohistochemical staining of MYBL2, CCL5, CD8⁺ T cells, and CD163⁺ tumor-associated macrophages in intrahepatic cholangiocarcinoma. Representative images of high and low expression of MYBL2, CCL5, CD8, and CD163 in tumor tissues. MYBL2 shows predominantly nuclear staining, whereas CCL5 is mainly localized in the cytoplasm of tumor cells. CD8⁺ T cells and CD163⁺ macrophages are mainly observed in the tumor stroma. Scale bars: 20 μm

Representative immunohistochemical staining demonstrated clear and specific expression of all four markers (Fig. 1). MYBL2 showed predominantly nuclear staining, whereas CCL5 was mainly expressed in the cytoplasm of tumor cells. CD8⁺ T cells and CD163⁺ macrophages were observed within the tumor nests and surrounding stromal regions, consistent with their expected distribution in the tumor immune microenvironment.

Baseline characteristics stratified by MYBL2 expression are summarized in Table 1. Most clinicopathologic variables were similarly distributed between MYBL2-Low and MYBL2-High groups, although MYBL2-High tumors showed lower rates of R0 resection (50.0% vs. 69.8%, *p* = 0.036) and fewer patients with elevated CA72-4 (14.1% vs. 30.2%, *p* = 0.048). Among the 70 patients who received adjuvant PD-1 therapy, the four MYBL2–CCL5 phenotypes exhibited comparable baseline features (Supplementary Table 1), suggesting that differences in survival and treatment response were not attributable to baseline imbalances within this cohort.

### Correlation among MYBL2, CCL5, and immune cell markers

**Fig. 2:**
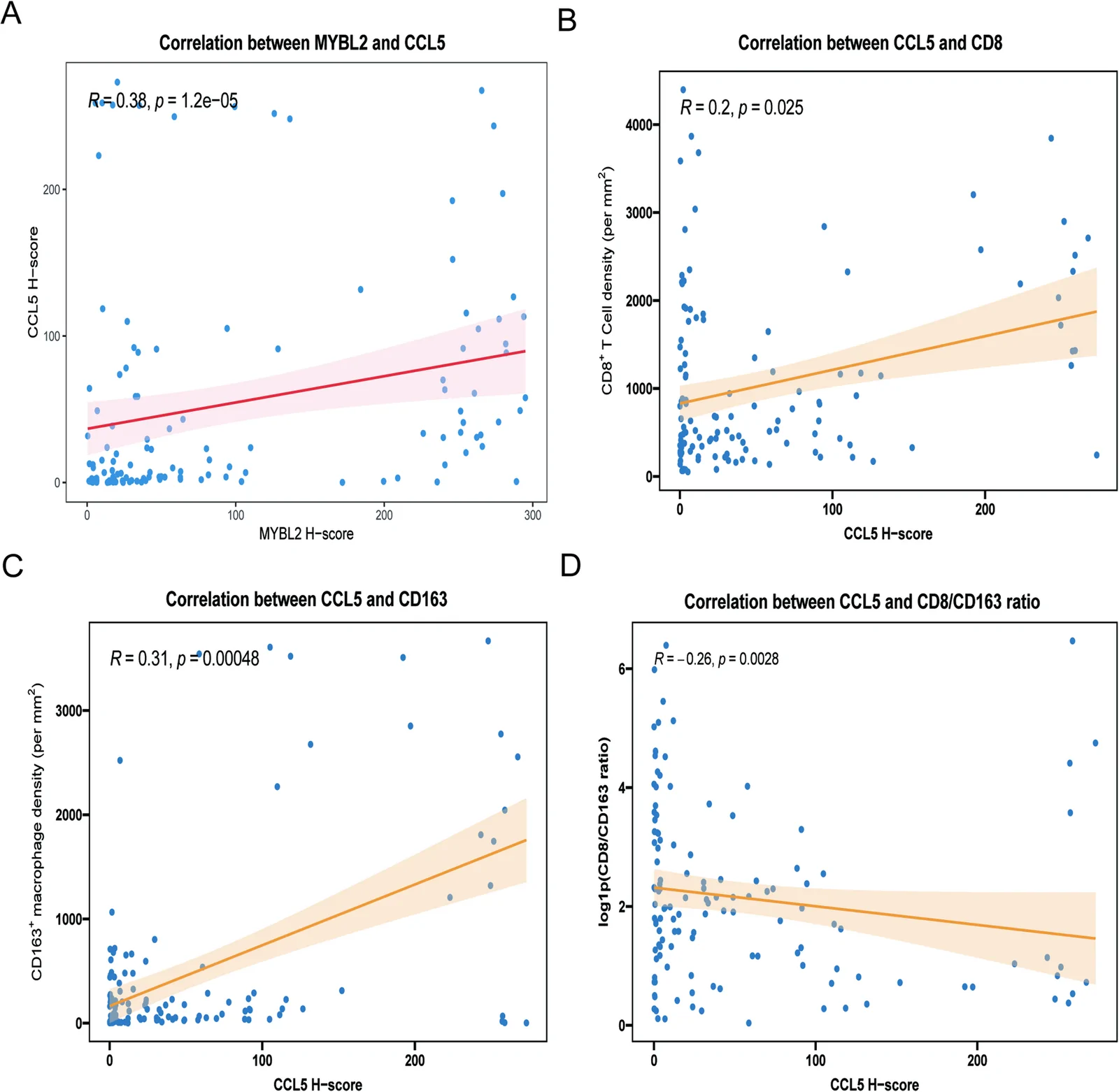
MYBL2–CCL5 co-expression is associated with TAM enrichment and a reduced CD8⁺/CD163⁺ratio in ICC. (**A**) Association between MYBL2 and CCL5 H-scores in ICC tumor tissues. (**B**) Association between CCL5 H-score and CD8⁺ T cell density (cells/mm²). (**C**) Association between CCL5 H-score and CD163⁺ macrophage density (cells/mm²). (**D**) Association between CCL5 H-score and the log-transformed CD8⁺/CD163⁺ ratio. Each dot represents an individual patient sample. Solid lines indicate locally estimated trend lines (LOESS), and shaded areas denote 95% confidence intervals. Correlation coefficients (R) and corresponding p values are shown in each panel

Correlation analysis showed a moderate positive correlation between MYBL2 and CCL5 (Fig. 2A, *r* = 0.38, *p* < 0.001). CCL5 expression had a weak but significant association with CD8⁺ T-cell density (Fig. 2B, *r* = 0.20, *p* = 0.025) and a stronger association with CD163⁺ macrophage infiltration (Fig. 2C, *r* = 0.31, *p* < 0.001). Higher CCL5 levels were also linked to a reduced CD8/CD163 ratio (Fig. 2D, *r* = − 0.26, *p* < 0.001), reflecting a shift toward an immunosuppressive profile. These findings suggest that while CCL5 is associated with CD8⁺ T-cell infiltration, it shows a relatively stronger association with CD163⁺ macrophage enrichment, consistent with a macrophage-dominant immune contexture.

### Prognostic significance of MYBL2 and CCL5 expression

**Fig. 3:**
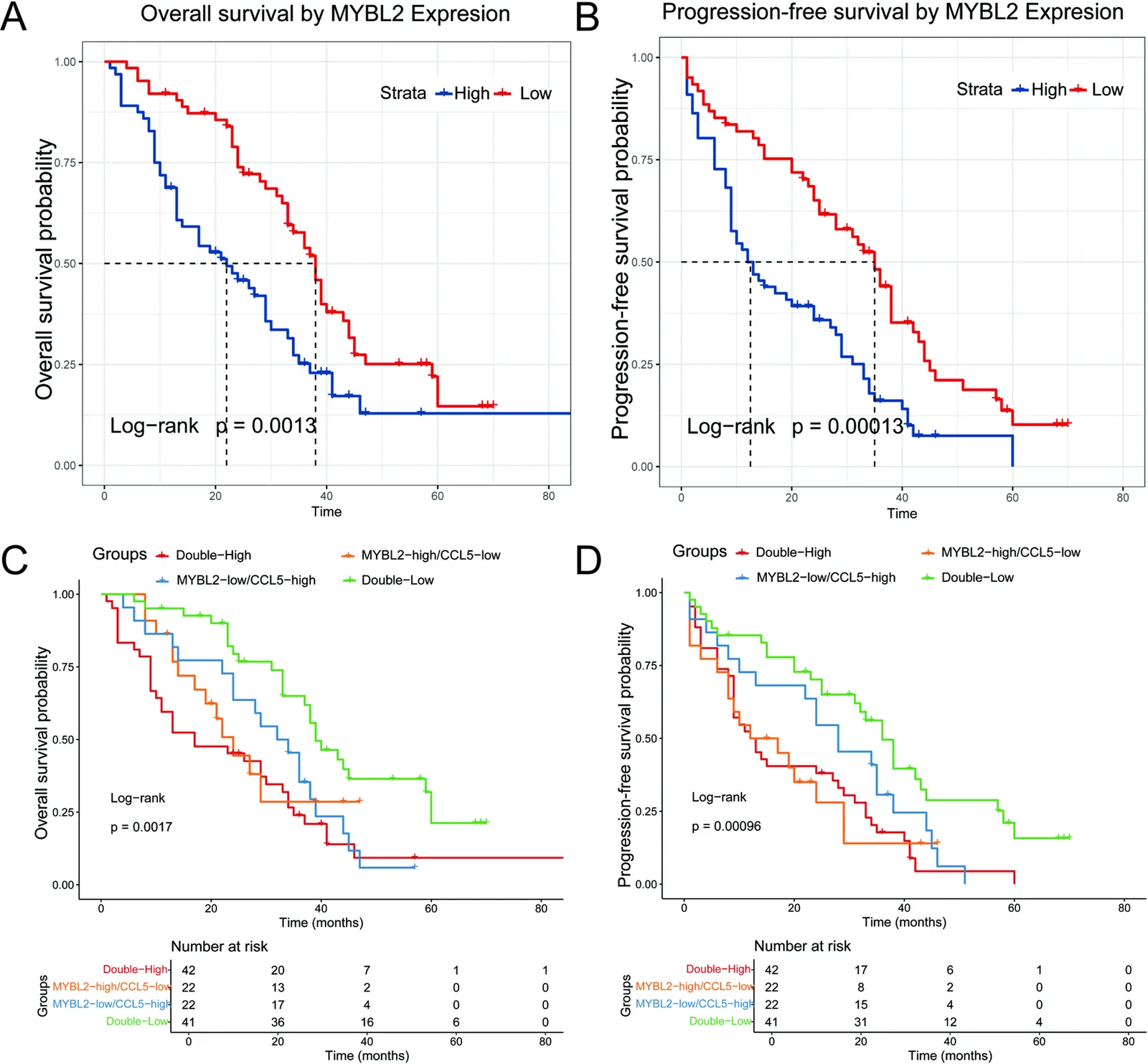
Survival outcomes according to MYBL2 expression and MYBL2–CCL5 co-expression phenotypes in ICC. (**A**) Kaplan–Meier analysis of overall survival (OS) stratified by MYBL2 expression (High vs. Low). (**B**) Kaplan–Meier analysis of progression-free survival (PFS) stratified by MYBL2 expression (High vs. Low). (**C**) Overall survival curves according to the four-group MYBL2–CCL5 expression classifier (Double-High, MYBL2-high/CCL5-low, MYBL2-low/CCL5-high, and Double-Low). (**D**) Progression-free survival curves according to the MYBL2–CCL5 classifier. Survival differences between groups were assessed using the log-rank test, with corresponding *p* values shown in each panel. Numbers at risk are displayed below the plots

Kaplan–Meier analyses showed that high MYBL2 expression was associated with significantly poorer OS and PFS (Fig. 3A–B; OS *p* = 0.0013, PFS *p* = 0.00013). When MYBL2 and CCL5 were combined into a four-group classifier, prognostic separation was more distinct than with either marker alone. The Double-High group had the worst survival, with a median overall survival (mOS) of 17 months (95% CI, 11–34), whereas the Double-Low group had the most favorable outcome, with an mOS of 39 months (95% CI, 37–60). The two discordant phenotypes showed intermediate risks, with an mOS of 24 months (95% CI, 17–NE) in the MYBL2-high/CCL5-low group and 33 months (95% CI, 24–44) in the MYBL2-low/CCL5-high group (Fig. 3C; OS *p* = 0.0017). When further classified into three groups, the mOS was 17 months (95% CI, 11–34) in the Double-High group, 28 months (95% CI, 22–38) in the Intermediate group, and 39 months (95% CI, 37–60) in the Double-Low group (log-rank *p* = 0.00054, Supplementary Fig. S1). For PFS, the Double-Low subgroup exhibited the most prolonged progression-free survival, followed by MYBL2-low/CCL5-high (Fig. 3D; log-rank *p* = 0.00096). Together, these results support MYBL2 as a prognostic marker and show that the MYBL2–CCL5 combination improves risk stratification by identifying a clear high-risk subgroup.

### Independent prognostic value

**Fig. 4:**
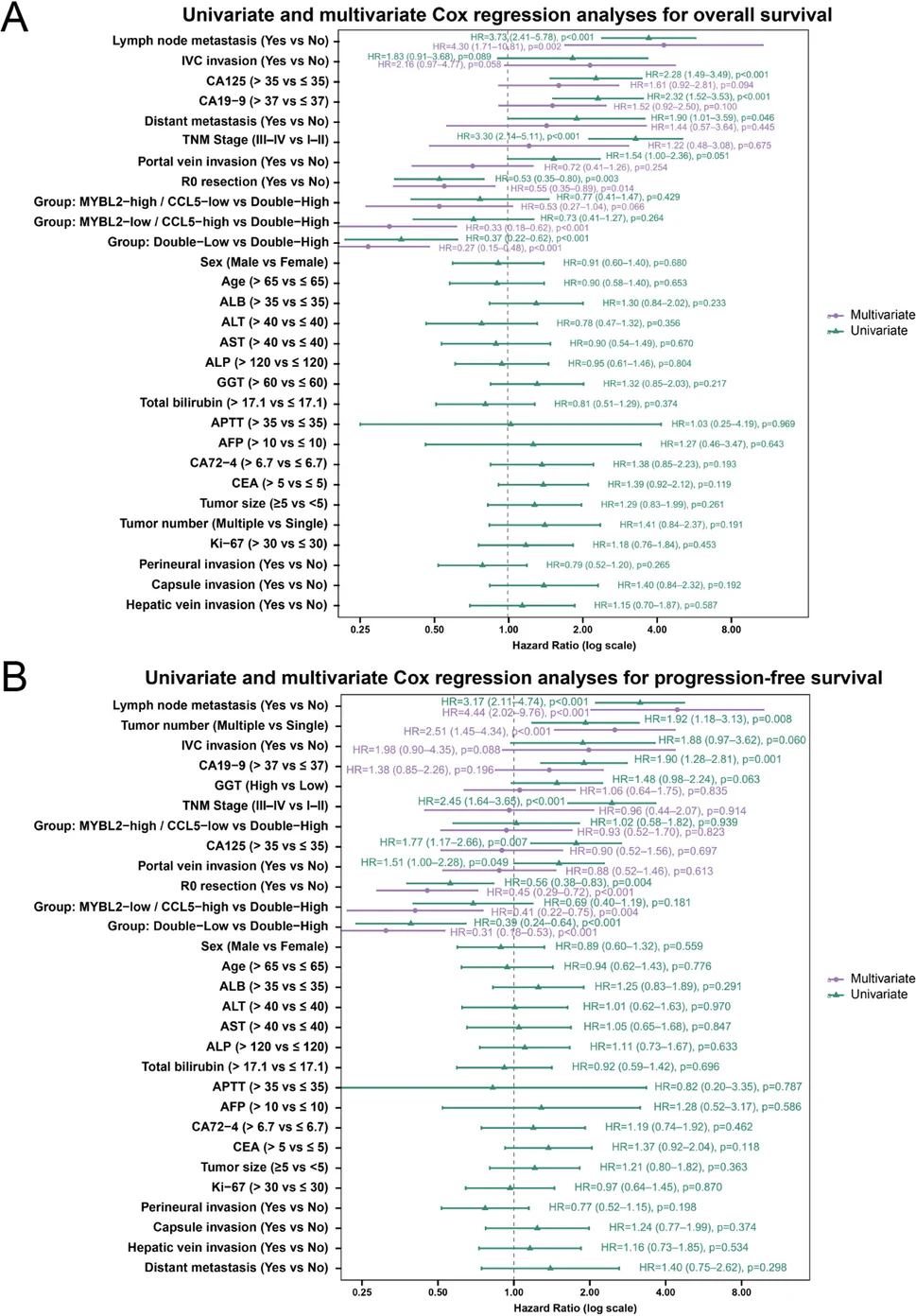
Univariate and multivariate Cox regression analyses for overall survival and progression-free survival in ICC. (**A**) Forest plot of univariate and multivariate Cox proportional hazards analyses for overall survival (OS). (**B**) Forest plot of univariate (all variables) and multivariate Cox analyses for progression-free survival (PFS), with variables meeting the pre-specified criterion (univariate *p* < 0.10) entered into the multivariate model

Univariate analysis showed that lymph-node metastasis, elevated CA19-9 and CA125, advanced TNM stage, distant metastasis, and the MYBL2–CCL5 classifier were associated with poorer OS, while R0 resection was protective. In the multivariable model, lymph-node metastasis remained an independent adverse factor, and R0 resection continued to improve survival (HR = 0.53, 95% CI 0.35–0.80, *p* = 0.003). The MYBL2–CCL5 classifier also retained strong prognostic value, with the Double-Low group showing a markedly lower mortality risk than Double-High tumors (HR = 0.27, 95% CI 0.15–0.48; Fig. 4A).

For PFS, univariate analysis identified lymph-node metastasis, IVC invasion, tumor number ≥ 2, elevated CA19-9 and CA125 levels, GGT elevation, portal vein invasion, R0 resection, and the MYBL2–CCL5 phenotype as significant predictors. In multivariable analysis, lymph-node metastasis and tumor multiplicity remained independently associated with shorter PFS, whereas R0 resection conferred a protective effect; notably, the MYBL2–CCL5 classifier retained its ability to stratify progression risk. Consistent with OS findings, the Double-Low group exhibited the most favorable PFS, with an approximately 70% lower risk of progression compared with the Double-High group (HR = 0.31, 95% CI 0.18–0.53; Fig. 4B).

Hazard ratios (HRs) are presented on a logarithmic scale with 95% confidence intervals. Results from univariate analyses are shown in green, and those from multivariate analyses are shown in purple. The MYBL2–CCL5 classifier is included as a categorical variable, with the Double-High group serving as the reference category.

### Predictive nomogram

**Fig. 5:**
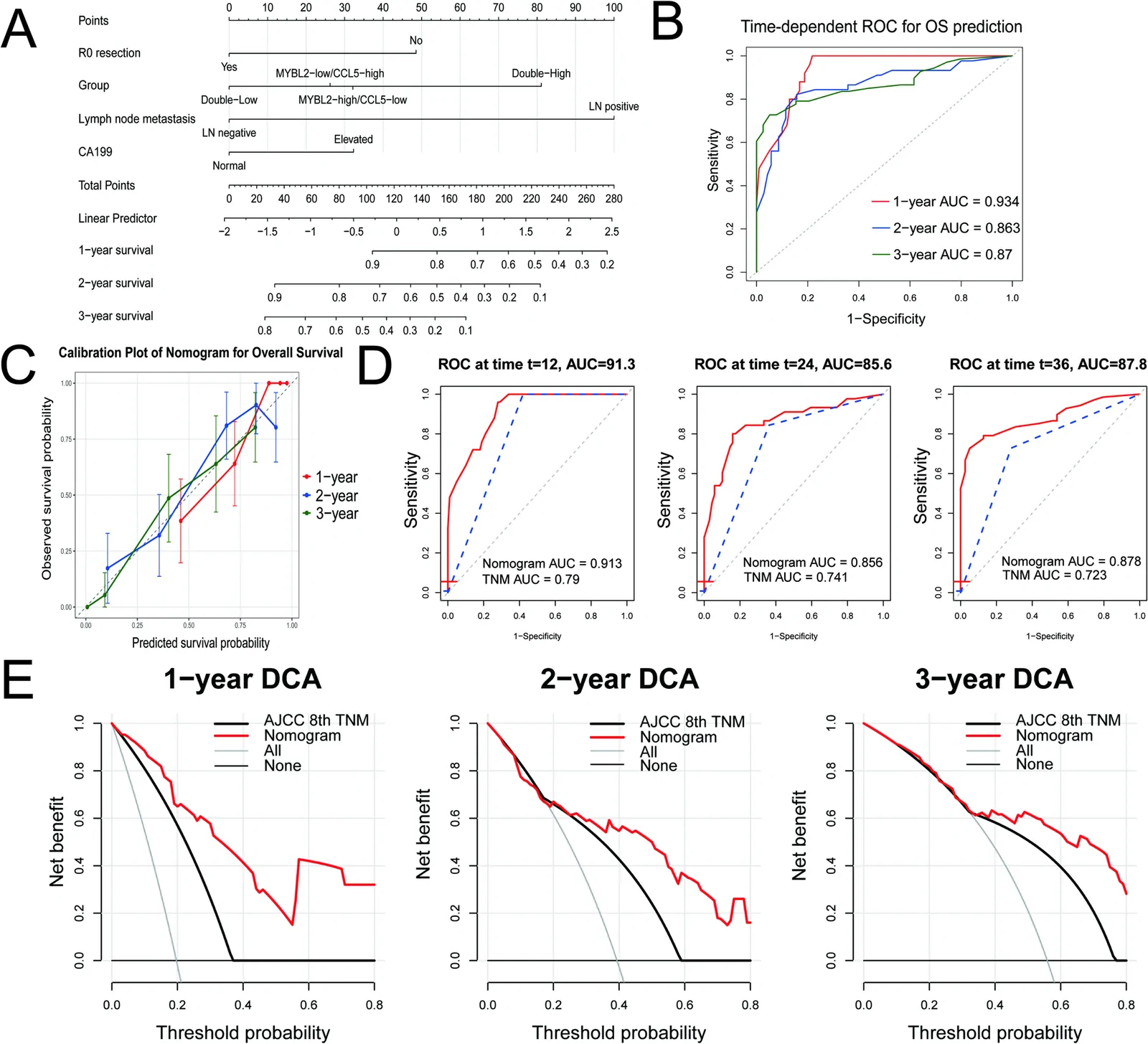
Construction and validation of the MYBL2–CCL5–based prognostic nomogram for overall survival in ICC. (**A**) Nomogram integrating independent prognostic factors derived from multivariate Cox analysis, including R0 resection status, the MYBL2–CCL5 classifier, lymph-node status, and CA19-9 level, to estimate 1-, 2-, and 3-year overall survival probabilities. (**B**) Time-dependent receiver operating characteristic (ROC) curves of the nomogram for predicting 1-, 2-, and 3-year overall survival. (**C**) Calibration plots of the nomogram showing agreement between predicted and observed overall survival probabilities at 1, 2, and 3 years. (**D**) Comparison of predictive accuracy between the nomogram and the AJCC 8th TNM staging system using time-dependent ROC curves at 1, 2, and 3 years. (**E**) Decision curve analysis (DCA) comparing the net clinical benefit of the nomogram with the AJCC 8th TNM staging system at 1-, 2-, and 3-year time points. The diagonal dashed line in calibration plots represents perfect agreement between predicted and observed outcomes. In ROC analyses, areas under the curve (AUCs) are indicated in each panel. In DCA, the horizontal line denotes the assumption that no patients experience the event, and the slanted line represents the assumption that all patients experience the event

Using the independent predictors of OS, we constructed a multivariable nomogram incorporating R0 resection status, the MYBL2–CCL5 classifier, lymph-node status, and CA19-9 level (Fig. 5A). The nomogram provides individualized 1-, 2-, and 3-year OS estimates and illustrates the relative contribution of each variable. It demonstrated good discrimination, with a C-index of 0.79 and a bootstrap-corrected C-index of 0.77. Time-dependent ROC curves confirmed strong predictive ability, with AUCs of 0.913, 0.856, and 0.878 at 1, 2, and 3 years (Fig. 5B).

Compared with the AJCC 8th TNM staging system, the nomogram showed consistently higher accuracy across all timepoints, particularly at 3 years (0.878 vs. 0.723; Fig. 5D). Calibration plots indicated close agreement between predicted and observed survival (Fig. 5C). Decision-curve analysis further suggested that the nomogram may provide greater net clinical benefit than AJCC staging across a range of threshold probabilities (Fig. 5E), indicating its potential value for postoperative risk stratification in ICC, pending further prospective validation.

### MYBL2–CCL5 phenotype and treatment response

**Fig. 6:**
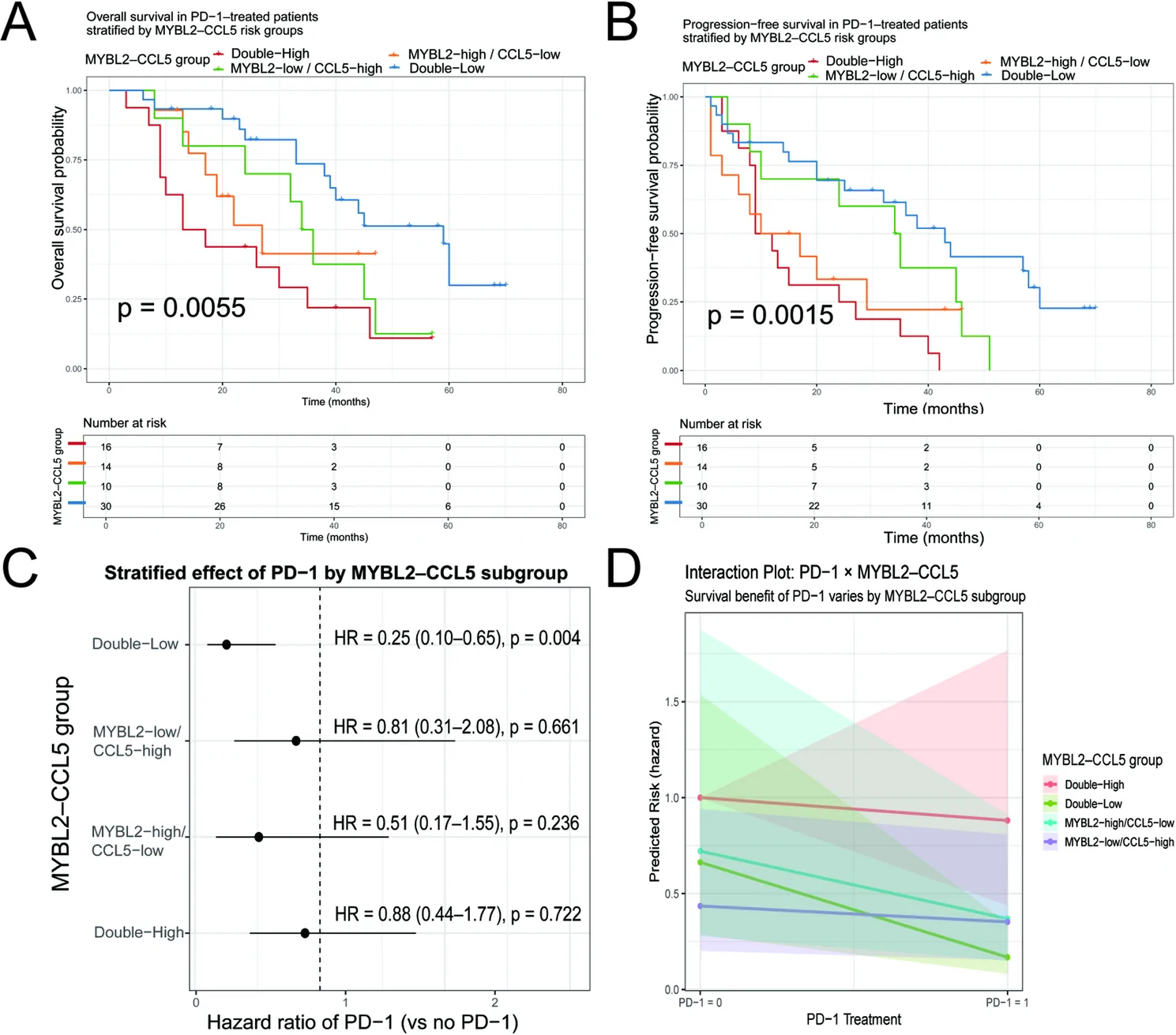
Differential clinical response to PD-1 therapy among MYBL2–CCL5 phenotypes. (**A**) Kaplan–Meier analysis of overall survival (OS) in patients treated with PD-1–based therapy, stratified by the four-group MYBL2–CCL5 classifier (Double-High, MYBL2-high/CCL5-low, MYBL2-low/CCL5-high, and Double-Low). (**B**) Kaplan–Meier analysis of progression-free survival (PFS) in PD-1–treated patients according to the MYBL2–CCL5 classifier. (**C**) Forest plot showing the stratified effect of PD-1 therapy on survival within each MYBL2–CCL5 subgroup, expressed as hazard ratios (HRs) comparing PD-1 treatment versus no PD-1 treatment. (**D**) Interaction plot illustrating the variation in predicted hazard according to PD-1 treatment status across MYBL2–CCL5 subgroups, indicating heterogeneity in treatment benefit. Log-rank *p* values are shown in panels A and B. In panel C, HRs are presented with 95% confidence intervals. Numbers at risk are displayed below Kaplan–Meier curves

To assess whether the MYBL2–CCL5 phenotype influences treatment response, we evaluated outcomes in patients who received PD-1 therapy (Fig. 6). Within this cohort, the classifier remained significantly associated with survival differences: the Double-High group had the poorest OS and PFS, whereas the Double-Low group showed the best outcomes, with the two discordant phenotypes falling in between (OS *p* = 0.013; PFS *p* = 0.003). Median overall survival was 15 months (95% CI, 9–NE) in the Double-High group, 27 months (95% CI, 17–NE) in the MYBL2-high/CCL5-low group, 35 months (95% CI, 24–NE) in the MYBL2-low/CCL5-high group, and 59 months (95% CI, 39–NE) in the Double-Low group.

Analysis within each subgroup demonstrated clear heterogeneity in PD-1 benefit. The Double-Low group showed the greatest hazard reduction with treatment, while the effect was attenuated in the discordant groups and minimal in the Double-High subgroup (Fig. 6C). Interaction testing confirmed a significant PD-1 × MYBL2–CCL5 effect (Fig. 6D), indicating that PD-1 benefit is largely confined to tumors with low-risk phenotypes. Together, these findings suggest that MYBL2–CCL5 co-expression was associated with prognosis and may also be associated with differential response patterns to PD-1 blockade, with Double-High tumors showing limited therapeutic response.

## Discussion

A key finding of this study was the identification of an ICC subgroup with concurrent MYBL2 and CCL5 upregulation. These tumors displayed a macrophage-dominant immune landscape and consistently poor outcomes, and the prognostic effect persisted after adjustment for conventional clinicopathologic factors, suggesting that the phenotype reflects an underlying biological program rather than simply tumor burden.

Of particular interest is the strong link between MYBL2–CCL5 co-expression and CD163⁺ macrophage accumulation. The degree of macrophage infiltration in Double-High tumors exceeded what would be expected based on stage alone, raising the possibility that MYBL2-related transcriptional activity may be associated with altered chemokine signaling and myeloid recruitment. Similar interactions between proliferative transcriptional programs and inflammatory signaling have been reported in other cancers, including MYBL2–CCL2 signaling in ovarian cancer and MYBL2-associated CCL15 signaling in bladder cancer [15, 16]. However, these studies do not provide direct evidence for a MYBL2–CCL5 regulatory axis. Therefore, the MYBL2–CCL5 association observed in the present study should be interpreted cautiously and regarded as hypothesis-generating. Further mechanistic studies are required to determine whether CCL5 is directly regulated by MYBL2 in ICC.

Another important aspect concerns treatment response. Among patients receiving PD-1 therapy, the Double-High subgroup showed little clinical benefit, with survival curves indicating minimal responsiveness. This aligns with clinical observations that macrophage-rich ICCs often respond poorly to immune checkpoint blockade [17, 18]. In several Double-High cases, radiologic progression occurred early despite sufficient therapeutic exposure, mirroring patterns reported in other macrophage-dominant hepatobiliary and pancreatic tumors [19–21]. Taken together, these clinical and biological findings suggest that MYBL2–CCL5 co-expression may be associated with limited benefit from PD-1 therapy.

The predictive performance of the MYBL2–CCL5–based nomogram was also notable. The model remained well calibrated and showed strong discrimination, outperforming TNM staging. This raises the possibility that immune composition may capture clinically relevant heterogeneity more effectively than anatomical extent alone. Although nomograms are increasingly common in oncology, few integrate immune features in a manner that may inform treatment stratification [22].

From a therapeutic perspective, the MYBL2–CCL5 phenotype suggests several potential interventions. If macrophage recruitment or polarization contributes to this phenotype, approaches such as CCR5 blockade, CSF1R inhibition, clodronate liposome–mediated macrophage depletion, or targeting the CCL5–CCR5 axis may warrant exploration in this subgroup [23–26]. Reprogramming macrophages toward a tumoricidal phenotype—via CD40 agonists, PI3Kγ inhibitors, or TREM2 blockade—has also shown encouraging preclinical activity [27–32]. Combining such macrophage-modulating strategies with PD-1 blockade may help overcome resistance and warrants further investigation [32–35].

Although these findings offer a framework for immunologic stratification, several issues remain unresolved. The mechanistic connection between MYBL2 and chemokine signaling requires experimental confirmation, and the influence of other immune cell populations on this subtype is not yet fully defined. In addition, the broader immunosuppressive context of the MYBL2–CCL5 Double-High group may also involve other suppressive immune populations, such as regulatory T cells (Tregs) and myeloid-derived suppressor cells (MDSCs), as well as checkpoint-related molecules including PD-L1, which were not systematically evaluated in the current study and warrant further investigation in future work. In addition, the retrospective design and limited sample size warrant caution when generalizing the results. Moreover, adjuvant PD-1 therapy was not assigned randomly, but was determined through multidisciplinary discussion based on high-risk pathological features and other clinical considerations. This non-random treatment allocation may have introduced selection bias, as patients receiving PD-1 therapy may have differed systematically from those who did not. Therefore, the findings regarding differential benefit from PD-1 therapy across MYBL2–CCL5 subgroups should be considered exploratory and interpreted with caution. Prospective studies incorporating detailed immune profiling, standardized treatment strategies, and treatment-response evaluation will be essential to validate the clinical utility of this classification.

## Conclusion

In summary, this study identifies a MYBL2–CCL5 co-expression phenotype in ICC that is characterized by a CD163⁺ macrophage–dominant, immunosuppressive microenvironment and an unfavorable CD8/CD163 balance. This phenotype was strongly associated with poor survival and remained an independent prognostic factor after adjustment for major clinicopathologic variables. Incorporating the MYBL2–CCL5 classifier into a prognostic model markedly improved risk stratification compared with conventional staging.

The phenotype also carried therapeutic implications. Double-High tumors appeared to derive limited benefit from PD-1 therapy, while patients with low expression of both markers had clearly better outcomes, suggesting that MYBL2–CCL5 status may help identify individuals less likely to respond to immunotherapy. The high prevalence of aggressive clinical features in our cohort may partly explain the larger proportion of Double-High cases and the limited immunotherapy response observed.

Overall, MYBL2–CCL5 co-expression emerges as both a prognostic biomarker and a potential therapeutic target in ICC. Prospective studies with mechanistic validation are needed to determine whether targeting MYBL2–CCL5–associated immune suppression—such as TAM-modulating or CCR5-directed strategies—can improve outcomes in this high-risk subgroup.

## Supplementary Information

Supplementary Material 1

Supplementary Material 2

## Data Availability

The datasets generated and analyzed during the current study are available from the corresponding author upon reasonable request.
