# Supplementary material for: Correlated expression of MYBL2 and CCL5 defines an immunosuppressive microenvironment and predicts poor prognosis in intrahepatic cholangiocarcinoma
Source: BMC Cancer. 2026 Jun 17;26:1053. doi: 10.1186/s12885-026-16219-4 (PMC13543640; doi:10.1186/s12885-026-16219-4)
Supplement: Supplementary file 1 [file 12885_2026_16219_MOESM2_ESM.pdf]

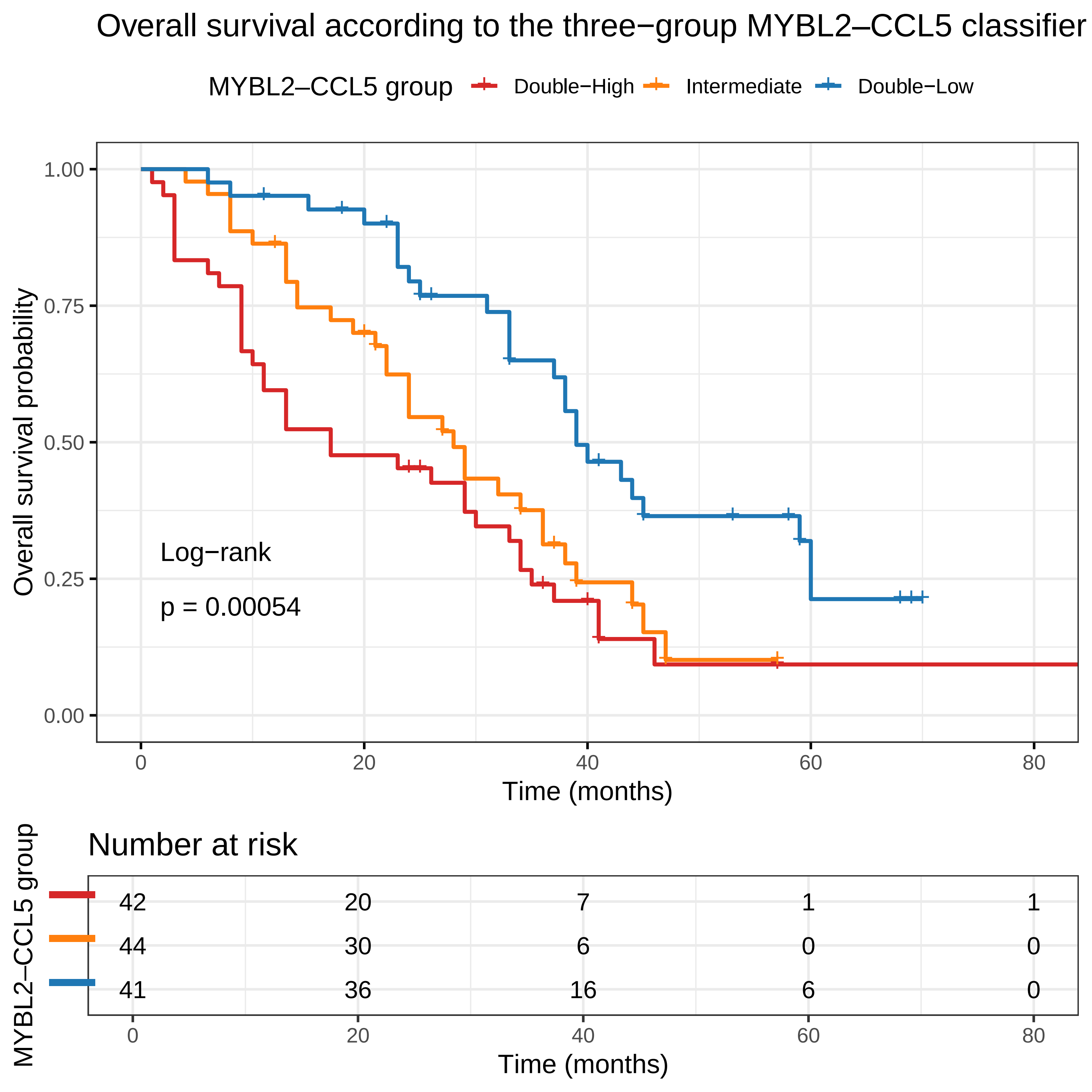
Overall survival according to the three−group MYBL2–CCL5 classifier
MYBL2–CCL5 group
Double−High
Intermediate
Double−Low
Overall survival probability
1.00
0.75
0.50
0.25
0.00
Log−rank
p = 0.00054
0
20
40
60
80
Time (months)
Number at risk
MYBL2–CCL5 group
42 20 7 1 1
44 30 6 0 0
41 36 16 6 0
0
20
40
60
80
Time (months)
