# Supplementary material for: Correlated expression of MYBL2 and CCL5 defines an immunosuppressive microenvironment and predicts poor prognosis in intrahepatic cholangiocarcinoma
Source: BMC Cancer. 2026 Jun 17;26:1053. doi: 10.1186/s12885-026-16219-4 (PMC13543640; doi:10.1186/s12885-026-16219-4)
Supplement: Supplementary file 2 [file 12885_2026_16219_MOESM1_ESM.docx]

Table 2. Clinicopathologic features of ICC patients according to combined MYBL2–CCL5 expression phenotypes.

|  | Double-High (N=16) | Double-Low (N=30) | MYBL2-high/CCL5-low (N=14) | MYBL2-low/CCL5-high (N=10) | Overall (N=70) | P value |
| --- | --- | --- | --- | --- | --- | --- |
| Sex |  |  |  |  |  | 0.547 |
| Female | 7 (43.8%) | 9 (30.0%) | 6 (42.9%) | 2 (20.0%) | 24 (34.3%) |  |
| Male | 9 (56.3%) | 21 (70.0%) | 8 (57.1%) | 8 (80.0%) | 46 (65.7%) |  |
| Age |  |  |  |  |  | 0.896 |
| ≤ 65 | 12 (75.0%) | 20 (66.7%) | 11 (78.6%) | 7 (70.0%) | 50 (71.4%) |  |
| > 65 | 4 (25.0%) | 10 (33.3%) | 3 (21.4%) | 3 (30.0%) | 20 (28.6%) |  |
| ALB |  |  |  |  |  | 0.752 |
| ≤ 35 | 10 (62.5%) | 20 (66.7%) | 11 (78.6%) | 6 (60.0%) | 47 (67.1%) |  |
| > 35 | 6 (37.5%) | 10 (33.3%) | 3 (21.4%) | 4 (40.0%) | 23 (32.9%) |  |
| ALT |  |  |  |  |  | 0.943 |
| ≤ 40 | 12 (75.0%) | 21 (70.0%) | 11 (78.6%) | 7 (70.0%) | 51 (72.9%) |  |
| > 40 | 4 (25.0%) | 9 (30.0%) | 3 (21.4%) | 3 (30.0%) | 19 (27.1%) |  |
| AST |  |  |  |  |  | 0.396 |
| ≤ 40 | 14 (87.5%) | 24 (80.0%) | 10 (71.4%) | 6 (60.0%) | 54 (77.1%) |  |
| > 40 | 2 (12.5%) | 6 (20.0%) | 4 (28.6%) | 4 (40.0%) | 16 (22.9%) |  |
| ALP |  |  |  |  |  | 0.408 |
| ≤ 120 | 13 (81.3%) | 19 (63.3%) | 9 (64.3%) | 5 (50.0%) | 46 (65.7%) |  |
| > 120 | 3 (18.8%) | 11 (36.7%) | 5 (35.7%) | 5 (50.0%) | 24 (34.3%) |  |
| GGT |  |  |  |  |  | 0.328 |
| ≤ 60 | 7 (43.8%) | 12 (40.0%) | 5 (35.7%) | 1 (10.0%) | 25 (35.7%) |  |
| > 60 | 9 (56.3%) | 18 (60.0%) | 9 (64.3%) | 9 (90.0%) | 45 (64.3%) |  |
| TBil |  |  |  |  |  | 0.925 |
| ≤ 17.1 | 11 (68.8%) | 22 (73.3%) | 11 (78.6%) | 7 (70.0%) | 51 (72.9%) |  |
| > 17.1 | 5 (31.3%) | 8 (26.7%) | 3 (21.4%) | 3 (30.0%) | 19 (27.1%) |  |
| APTT |  |  |  |  |  | 0.551 |
| ≤ 35 | 16 (100%) | 28 (93.3%) | 14 (100%) | 9 (90.0%) | 67 (95.7%) |  |
| > 35 | 0 (0%) | 2 (6.7%) | 0 (0%) | 1 (10.0%) | 3 (4.3%) |  |
| AFP |  |  |  |  |  | 0.056 |
| ≤ 10 | 16 (100%) | 30 (100%) | 12 (85.7%) | 10 (100%) | 16 (100%) |  |
| > 10 | 0 (0%) | 0 (0%) | 2 (14.3%) | 0 (0%) | 2 (2.9%) |  |
| CA199 |  |  |  |  |  | 0.685 |
| ≤ 37 | 6 (37.5%) | 17 (56.7%) | 7 (50.0%) | 5 (50.0%) | 35 (50.0%) |  |
| > 37 | 10 (62.5%) | 13 (43.3%) | 7 (50.0%) | 5 (50.0%) | 35 (50.0%) |  |
| CA125 |  |  |  |  |  | 0.224 |
| ≤ 35 | 12 (75.0%) | 25 (83.3%) | 8 (57.1%) | 6 (60.0%) | 51 (72.9%) |  |
| > 35 | 4 (25.0%) | 5 (16.7%) | 6 (42.9%) | 4 (40.0%) | 19 (27.1%) |  |
| CA724 |  |  |  |  |  | 0.065 |
| ≤ 6.7 | 15 (93.8%) | 25 (83.3%) | 11 (78.6%) | 5 (50.0%) | 56 (80.0%) |  |
| > 6.7 | 1 (6.3%) | 5 (16.7%) | 3 (21.4%) | 5 (50.0%) | 14 (20.0%) |  |
| CEA |  |  |  |  |  | 0.157 |
| ≤ 5 | 14 (87.5%) | 17 (56.7%) | 8 (57.1%) | 6 (60.0%) | 45 (64.3%) |  |
| > 5 | 2 (12.5%) | 13 (43.3%) | 6 (42.9%) | 4 (40.0%) | 25 (35.7%) |  |
| Tumor Size |  |  |  |  |  | 0.789 |
| ≤ 5 | 6 (37.5%) | 9 (30.0%) | 5 (35.7%) | 2 (20.0%) | 22 (31.4%) |  |
| > 5 | 10 (62.5%) | 21 (70.0%) | 9 (64.3%) | 8 (80.0%) | 48 (68.6%) |  |
| Tumor Number |  |  |  |  |  | 0.730 |
| Single | 14 (87.5%) | 25 (83.3%) | 12 (85.7%) | 7 (70.0%) | 58 (82.9%) |  |
| Multiple | 2 (12.5%) | 5 (16.7%) | 2 (14.3%) | 3 (30.0%) | 12 (17.1%) |  |
| Ki67 |  |  |  |  |  | 0.132 |
| ≤30 | 5 (31.3%) | 11 (36.7%) | 8 (57.1%) | 1 (10.0%) | 25 (35.7%) |  |
| >30 | 11 (68.8%) | 19 (63.3%) | 6 (42.9%) | 9 (90.0%) | 45 (64.3%) |  |
| LN Metastasis |  |  |  |  |  | 0.086 |
| No | 7 (43.8%) | 20 (66.7%) | 4 (28.6%) | 4 (40.0%) | 35 (50.0%) |  |
| Yes | 9 (56.3%) | 10 (33.3%) | 10 (71.4%) | 6 (60.0%) | 35 (50.0%) |  |
| Perineural Invasion |  |  |  |  |  | 0.845 |
| No | 8 (50.0%) | 11 (36.7%) | 5 (35.7%) | 4 (40.0%) | 28 (40.0%) |  |
| Yes | 8 (50.0%) | 19 (63.3%) | 9 (64.3%) | 6 (60.0%) | 42 (60.0%) |  |
| Capsule Invasion |  |  |  |  |  | 0.734 |
| No | 2 (12.5%) | 6 (20.0%) | 3 (21.4%) | 3 (30.0%) | 14 (20.0%) |  |
| Yes | 14 (87.5%) | 24 (80.0%) | 11 (78.6%) | 7 (70.0%) | 56 (80.0%) |  |
| R0 Resection |  |  |  |  |  | 0.512 |
| No | 7 (43.8%) | 11 (36.7%) | 7 (50.0%) | 2 (20.0%) | 27 (38.6%) |  |
| Yes | 9 (56.3%) | 19 (63.3%) | 7 (50.0%) | 8 (80.0%) | 43 (61.4%) |  |
| Portal Invasion |  |  |  |  |  | 0.967 |
| No | 11 (68.8%) | 20 (66.7%) | 9 (64.3%) | 6 (60.0%) | 46 (65.7%) |  |
| Yes | 5 (31.3%) | 10 (33.3%) | 5 (35.7%) | 4 (40.0%) | 24 (34.3%) |  |
| IVC Invasion |  |  |  |  |  | 0.347 |
| No | 15 (93.8%) | 26 (86.7%) | 14 (100%) | 8 (80.0%) | 63 (90.0%) |  |
| Yes | 1 (6.3%) | 4 (13.3%) | 0 (0%) | 2 (20.0%) | 7 (10.0%) |  |
| Hepatic vein Invasion |  |  |  |  |  | 1 |
| No | 12 (75.0%) | 22 (73.3%) | 10 (71.4%) | 8 (80.0%) | 52 (74.3%) |  |
| Yes | 4 (25.0%) | 8 (26.7%) | 4 (28.6%) | 2 (20.0%) | 18 (25.7%) |  |
| Distant Metastasis |  |  |  |  |  | 0.403 |
| No | 14 (87.5%) | 26 (86.7%) | 14 (100%) | 8 (80.0%) | 62 (88.6%) |  |
| Yes | 2 (12.5%) | 4 (13.3%) | 0 (0%) | 2 (20.0%) | 8 (11.4%) |  |
| TNM Stage |  |  |  |  |  | 0.300 |
| Stage I–II | 6 (37.5%) | 18 (60.0%) | 6 (42.9%) | 3 (30.0%) | 33 (47.1%) |  |
| Stage III–IV | 10 (62.5%) | 12 (40.0%) | 8 (57.1%) | 7 (70.0%) | 37 (52.9%) |  |
